# The effect of recombinant human thrombopoietin (rhTPO) on sepsis patients with acute severe thrombocytopenia: a study protocol for a multicentre randomised controlled trial (RESCUE trial)

**DOI:** 10.1186/s12879-019-4388-2

**Published:** 2019-09-06

**Authors:** Zhigang Zhou, Tienan Feng, Yun Xie, Peijie Huang, Hui Xie, Rui Tian, Biyun Qian, Ruilan Wang

**Affiliations:** 1 Department of Critical Care Medicine, Shanghai General Hospital of Nanjing Medical University, 650# New Songjiang Road, Songjiang, Shanghai, 201620 China; 2 Clinical Research Institute, Shanghai Jiaotong University School of Medicine, 227# South Chongqing Road, Xuhui, Shanghai, 200025 China

**Keywords:** Sepsis, Thrombopoietin, Severe thrombocytopenia, Mortality, Rescue therapy

## Abstract

**Background:**

Sepsis is still a common critical disease with high morbidity and mortality in intensive care unit. Despite published guidelines for sepsis, development of antibiotic therapy and advanced organ support technologies, the mortality of sepsis patients is still 25% or more. It is necessary to distinguish the subtypes of sepsis, and the targeted therapy for the patients need to be explored. Platelets have various biological functions in hemostasis and thrombosis, host defense, inflammatory/immune responses and tissue repair/regeneration. Moreover, severe thrombocytopenia or sustained thrombocytopenia was closely associated with multiply organ dysfunction and higher mortality in sepsis patients. The clinical therapies for thrombocytopenia are platelet transfusion and platelet-elevating drugs. However, platelet transfusion has many defects in clinical practice in sepsis patients, and the impact of platelet-elevating drugs for sepsis patients is still unclear. RESCUE trial is aim to explore the effect of a platelet-elevating drug, recombinant human thrombopoietin (rhTPO), as an effective rescue therapy on sepsis patients with acute severe thrombocytopenia.

**Methods:**

It is a randomized, open-label, multi-center, controlled trial in 5 tertiary academic hospitals including medical, surgical or general ICUs. In this study, a total of 200 sepsis patients with severe thrombocytopenia will be randomly assigned in a 1:1 ratio to the control and rhTPO group. The patients will be followed up to 28 days after randomization. All patients in two groups receive the same treatment based on the guideline of Surviving Sepsis Campaign. Primary outcome is 28-day mortality. Secondary outcomes are the changes of PCs, blood transfusion, biomarkers of infection and organ function, days free from advanced organ support, drug-related adverse events, the length of ICU and hospital stay.

**Discussion:**

RESCUE trial is the first randomized controlled trial to explore the impact of rhTPO for severe thrombocytopenia in sepsis patients diagnosed by sepsis-3.0 standard. Furthermore, RESCUE trial results will be of significant clinical value on the targeted therapy and add clinical evidence that rhTPO is an effective rescue therapy for these sepsis patients.

**Trial registration:**

ClinicalTrials.gov: NCT02707497. Registered Date: March 3rd, 2016. Protocol Version 3. Protocol Date: January 25th, 2019.

## Background

Sepsis defined as infection-induced organ dysfunction is a common critical disease with high mortality and is the leading cause of death in intensive care unit [1, 2]. Furthermore, the incidence of sepsis in ICU is increasing year by year, reaching more than 30% [3–5]. In recent decades, despite published guidelines for sepsis, rapid development of antibiotic therapy and advanced organ support technologies, the mortality of sepsis patients is still 25% or more [6]. There have been many large RCT clinical studies targeting to improve the prognosis of sepsis [7–10]. Unfortunately, these studies all had negative results and no therapy have been found to improve clinical outcomes in sepsis patients. It could be ascribed to that sepsis is a critical disease caused by many different etiologies which is involved in different pathophysiological changes. Besides, in most past studies, sepsis patients diagnosed by the standards with poor specificity had poor homogeneity. Therefore, to improve clinical outcomes of sepsis patients, distinguishing different subtypes of sepsis and developing targeted therapy for the certain subtypes need to be explored.

Platelet counts (PCs) are frequently altered in patients with sepsis, and are implicated in the pathogenesis of multi-organ failure. Thrombocytopenia is common in sepsis patients, the incidence of which is even as high as 55% [11]. There exist the following mechanisms about sepsis-related thrombocytopenia [12, 13]:1) Some toxins and inflammatory mediators induced by sepsis can inhibit the formation and release of megakaryocytes in bone marrow to cause thrombocytopenia; 2) Some toxins and inflammatory mediators induced by sepsis can directly destroy platelets; 3) Sepsis is often accompanied with local microthrombosis or even cause disseminated intravascular coagulation (DIC) to result in massive platelet depletion; 4) Thrombocytopenia can be induced by some clinical treatments for sepsis patients such as renal replace therapy (RRT), heparin and antibiotics. Until now, it has been confirmed that platelets have various biological functions, not only in hemostasis and thrombosis but also in the host defense, inflammatory/immune responses, tissue repair and regeneration [14–16]. Clinically, thrombocytopenia was closely associated with multiply organ dysfunction and the higher mortality in sepsis patients [17, 18]. Moreover, a multicenter prospective trial in France pointed out that thrombocytopenia was an early prognostic indicator for sepsis patients, and the occurrence and duration of thrombocytopenia were closely related to length of ICU stay, hospitalization stay and 28-day mortality. Furthermore, the greater the extent of thrombocytopenia was, the higher the mortality was [19]. Another study found that severe thrombocytopenia released more inflammatory factors, stimulated endothelial cells, inhibited leukocyte adhesion/activation related signaling pathways, activated complement related signaling pathways, and caused dysregulated host defense responses. Furthermore, 30-day mortality of sepsis patients with severe thrombocytopenia was more than 50% [20]. In our previous meta-analysis study, we also found that thrombocytopenia was associated with poor prognosis and increased the complication including shock, bleeding and acute kidney injury in sepsis patients [21]. Besides, the patients who had sustained thrombocytopenia or a drop-in PCs of > 30% during ICU had the higher mortality [22]. The longer the duration of thrombocytopenia is, the worse the prognosis of these patients have, and rapid recovery of PCs may reduce the mortality of these patients. In our single-center, retrospective cohort study, we found that sepsis patients with severe thrombocytopenia might have the more blood product transfusion, the shorter days free from organ support, the shorter survival days and the higher 28-day mortality. Therefore, there is an urgent need to explore a safe, effective therapy for recovery of PCs in sepsis patients with thrombocytopenia, especially severe thrombocytopenia.

The most common clinical therapy to increase PCs is platelet transfusion. Theoretically, one standard unit dose of platelet transfusion can increase PCs by 20 X10^9^/L. However, because of resource scarcity, transfusion-related immune and infectious complications, ineffective transfusion and platelet antibody production, platelet transfusion is limited in clinical practice and has a rigorous indication in sepsis patients [6, 23]. So, platelet-elevating drugs probably as the rescue therapy improve the prognosis of sepsis patients with thrombocytopenia through recovering PCs.

Thrombopoietin as a highly specific platelet-stimulator can directly act on bone marrow hematopoietic stem cells and bind to its specific receptor Mpl. TPO can stimulate all stages of megakaryocyte formation and specifically increase PCs [24, 25]. In addition, some studies showed that TPO improved T lymphocyte function, regulated the release of inflammatory mediators, reduced endothelial cell injury, decreased platelet aggregation and consumption [26]. Recombinant human thrombopoietin (rhTPO) is a full-length glycosylated-TPO produced by Chinese hamster ovary cells, which has the similar biological functions as endogenous TPO. It has been confirmed that rhTPO could effectively increase PCs in patients with immune or chemotherapy-related thrombocytopenia and has no serious adverse effects [27–29]. Recently, Kong Z et al. reported that rhTPO effectively increased PCs in pregnancy patients with immune thrombocytopenia and had no adverse effects, which suggests that rhTPO is a safe drug [30]. However, to date, there are very few studies about the impact of rhTPO for sepsis patients, especially prospective RCT study. Meanwhile, the appropriate patients, using course and safety of rhTPO in sepsis are also still unclear. In our previous retrospective study, for patients with severe thrombocytopenia, rhTPO could rapidly increase PCs, reduce 28-day mortality, and there were no severe adverse events during rhTPO treatment. However, there were several limitations in the study, which was a retrospective, single-center study; the sample size was small and the patients were non-randomized. So, the purpose of this study is to confirm the clinical effect of rhTPO on sepsis patients with severe thrombocytopenia.

## Methods and design

### Objectives

The objective of this study is to determine whether rhTPO as an effective and safe rescue therapy rapidly increases the platelets counts, shortens recovery time of platelets, reduces platelets transfusion and bleeding events, and improves the prognosis in sepsis patients with severe thrombocytopenia.

### Study design and setting

The study is designed as a randomized, open-label, multi-center, controlled study in 5 tertiary academic medical centers which are medical, surgical or general ICUs. Professor RL. W (Shanghai General Hospital, China) will coordinate all the operational processes and Professor QB. Y (Shanghai Jiao Tong University School of Medicine, China) will conduct all the statistical analyses. Eligible patients who give their consent to participate in the study will be randomly assigned in a 1:1 ratio to treat with rhTPO (rhTPO group) or without any platelet-elevating drugs (control group). Both two groups receive appropriate medical treatment according to the current guideline of Surviving Sepsis Campaign (SSC) [6], and will be assessed for the outcomes at baseline, after the intervention and at 28-day follow-up. The end of the study is defined by the last follow-up of the last enrolled patient.

Patients in rhTPO group will be treated with rhTPO (TPIAO™, Shenyang Sunshine Pharmaceutical Co., Ltd. Shengyang, China) at a dose of 15000u/d, subcutaneous injection, for 7 consecutive days. RhTPO in the study is for free. The time from randomization to administration of rhTPO will be within 24 h. The drug will be terminated when PCs reach the standard of clinical recovery of platelets (PCs ≥100 × 10^9^/L or the increasing of PCs ≥ 50 × 10^9^/L lasting for at least 3 days) or severe adverse effects happened. The clinical recovery time of platelets was defined as the time it takes to reach clinical recovery. Patients in control group will not be treated with any platelet-elevating drugs.

In both control and rhTPO groups, there is the same standard of platelet transfusion: when PCs are below 10 × 10^9^/L in the absence of apparent bleeding; or below 20 × 10^9^/L if the patient has a significant risk of bleeding; or below 50 × 10^9^/L if the patient has active bleeding or need invasive operation. During the study, any other platelet-elevating drugs will be forbidden.

Drug-associated serious adverse events should be reported to Shanghai General Hospital within 24 h of participating site study staff becoming aware of the occurrence, and a member of Shanghai General Hospital will be on call 24 h a day via telephone. Serious adverse events are defined as any unexpected medical occurrence that meets one of more of the following criteria: 1) Results in death; 2) Is life-threatening; 3) Results in persistent or significant disability/incapacity. If the patient’s injury is directly induced by rhTPO, the cost of the treatment will be covered by the researchers.

### Study population/ participants

A principle investigator (PI), assisted by a well-trained Co-PI in each clinical center, is responsible for selecting eligible patients based on the eligibility criteria and authorizing the staff to have the patients’ consents. Patients are eligible to be enrolled the study based on the following inclusion and exclusion criteria. Inclusion criteria: 1) age ≥ 18 years, and ≤ 75 years, no limitation of gender; 2) Diagnosis or clinical diagnosis of infection; 3) The change of Sequential Organ Failure Assessment (ΔSOFA) ≥ 2; 4) Platelets counts (PCs) < 50 × 10^9^/L; 5) Informed consent. Exclusion criteria: 1) History of the treatments with chemotherapeutic drugs or heparin drugs within six months; 2) History of bone marrow stem cell disorders, malignancy, or immunologic diseases; 3) History of bone marrow, lung, liver, kidney, pancreas, or small bowel transplantation; 4) Confirmed End-stage renal failure (GFR<10 ml/min, Scr>707 μmol/L); 5) Confirmed Disseminated Intravascular Coagulation (DIC); 6) Confirmed Hemorrhagic brain injury or need craniocerebral operation; 7) Died anticipated within 24 h; 8) Pregnant or lactating patients.

### Sample size

The 28-day all-cause mortality of sepsis patients varies in different patient populations. According to the previous studies, the 28-day mortality of sepsis patients with severe thrombocytopenia was more than 50% [19, 20, 31]. Furthermore, in our single-center, retrospective, cohort study, the 28-day mortality of these patients treated with rhTPO was 29.2%. Our sample size was calculated to detect a 20% difference (rhTPO group 30% Vs Control group 50%) in 28-day mortality. Based on a two-tailed test of two independent means, with a significance (α) level of 0.05 and 80% power, a minimum of 91 patients in each group for a total sample of 182 patients was required. It was assumed that the loss rate of this study is no more than 10%, and the minimum sample of this study was 100 patients in each group for a total sample of 200 patients.

### Randomization

Eligible patients will be randomly assigned to the control and rhTPO add-on treatment in a dynamic random and competitive design in clinical trial sites. Sequential organ failure assessment (SOFA), Acute Physiology and Chronic Health Evaluation II (APACHE II) scores are as the dynamic equilibrium factors. Randomization will be done after the first assessment, ensuring that the assessing occupational therapist will not be biased at this time by knowing the group assignment. A random number will be generated before the start of this study. The random number list will be kept by the third party and uploaded into a random data assigned system. When a patient is recruited in this study, the patient will then be assigned a random number by the system with corresponding treatment. If a patient is later excluded for any reason, the patient’s position in the randomization list will not be replaced by any new patient. Patients will be recruited consecutively. After forty patients have been recruited, the dynamic arithmetic will be introduced. Then, the random number assigned by the system will based on the equilibrium of dynamic factors.

### Blinding

No blinding will take place in this study. Eligible patients who have given informed consent will be randomized into either of the two groups.

### Outcome measures

#### Primary outcomes

Primary outcome of this study is the 28-day all-cause mortality.

#### Secondary outcomes

Secondary outcomes of this study have been seen as follow: 1) The changes of platelets counts (PCs) in the first 7 days; 2) The clinical recovery time of PCs; 3) The amount and proportion of blood transfusion (including platelets, RBC, Frozen plasma); 4) The changes of biomarkers of infection including procalcitonin, C-reactive protein (CRP), endotoxin; 5) The changes of the markers of organ function including cardiac, hepatic, renal, coagulation function; 6) The days free from advanced organ support (including vasoactive drugs/mechanical ventilation/renal replacement therapy) at 28 days; 7) The incidence of bleeding event; 8) The incidences of drug-related adverse events; 9) The length of ICU and hospital stay.

### Data collection

**Fig. 1:**
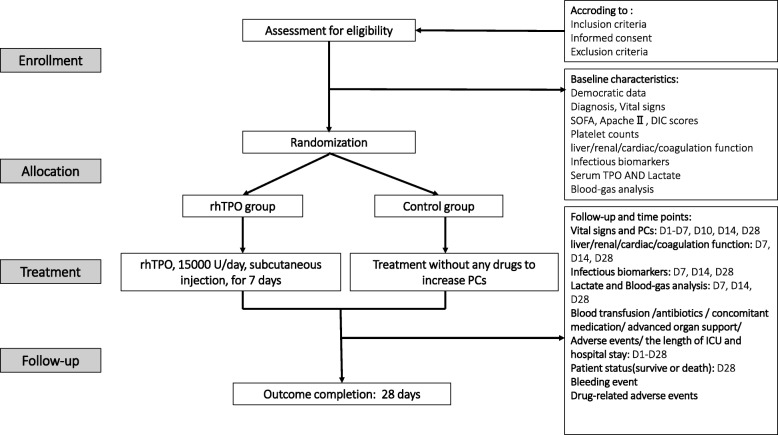
Flowchart of RESCUE trial procedure

A case report form (CRF) will be used for all patient who met the enrollment criteria to collect the data. Patients will be followed up to 28 days after randomization. The data will be abstracted from the patients’ electronic medical records: age, ethnicity, gender, patients’ type (medical/surgical), co-comorbidities, sites and pathogens of infection. Then, clinical characteristic baseline and laboratory results were tested within the first 24 h after randomization, including markers of organ functions and biomarkers of infection (Procalcitonin, C-reactive protein, Endotoxin). The severity of disease scores will be recorded, including APACHE II, SOFA, and DIC scores [32] in the first 24 h. The basic serum TPO level in patients will be tested by ELISA Kit (R&D Systems, Minneapolis, MN, USA). The clinical data will be tested in the point-in-time followed by protocols on the day of enrollment and periodically thereafter until the time of discharge or death, including the numbers of blood products transfusion (including platelets), antibiotics, concomitant medication, days free from advanced organ support, adverse events and the length of ICU or hospital stay. And if several clinical data or measurements will be performed at one day, the lowest/worst data will be retained. Patient status (survive or death) at the 28 days will be recorded through chart review or contact patients if necessary, and the major causes of death will be recorded if the patients will be died. Flowchart of this study procedure can be seen in Fig. 1.

### Data management and monitor

The data in the study will be managed by clinical trial staffs who will be authorized by the site PI at the sites. The site PI will be responsible for ensuring the accuracy, completeness, legibility, and timeliness of the data reported in the site. Researchers were not permitted to interpret records, and other researchers who are not involved in the patients’ treatment will assess the outcomes. Furthermore, to ensure inter-site reliability, the PIs will explicate operations manual (including the definitions for CRFs and guidelines for collecting data) before data collection and frequent communications among PIs will be kept through e-mail and WeChat during the trial.

Clinical Research Institute of Shanghai Jiao Tong University Medicine School will be responsible for the clinical data management. The Electronic Data Capture (EDC) system will be supplied by Edetek Inc. (Princeton, NJ, USA). All patients’ data will be recorded on paper CRFs and then enter into EDC system. Data in the EDC system will be checked by built-in algorithm, monitored when some modification is required, and traceable to source data. The trial sponsor (Professor RL. W, Shanghai General Hospital, China) have access to the final trial dataset.

Data and Safety Monitoring Committee (DSMC), independent from the coordinating centers and investigators, will perform an ongoing review of predefined safety parameters and overall study conduct. The DSMC established by Clinical Research Institutes of Shanghai General Hospital and Shanghai Jiao Tong University School of Medicine will be comprised of experts in clinical trials, biostatistics and intensive care medicine.

On all study-specific documents, other than the signed consent, the participant will be referred to by the study code, not by name. All study-specific documents at all centres including source document verification, informed consents, data quality, study drug reconciliation and the table of follow-up will be monitored by DSMC on several occasions during the study, independent from investigators and the sponsor. Besides, the study may also be audited by local or national regulatory authorities.

The study may be terminated or suspended at any time at the following reasons: 1) the request of the study management committee or a regulatory authority; 2) Funding or management problems; 3) Drug-associated severe adverse events. And Clinical Research Institute of Shanghai General Hospital can make the final decision to terminate the trial and will supply reason(s) for the termination or suspension to all the centers. Otherwise, the study is considered terminated upon completion of all patient treatments and evaluations. When discontinuing the trial, all records shall be kept for check.

### Statistical methods

Analyses will be conducted on an intention-to-treat and per-protocol population basis. Data for each assessment will be summarized for each treatment group and the descriptive statistics will be calculated depending on the data distribution for each assessment. Outcomes will be collected at the times defined in the study protocol. The primary efficacy variable will be the change in 28-day mortality. The change in 28-day mortality between the two groups will be analyzed by using Chi-Square. The proportions will be analyzed using logistic regression including baseline as covariate and group as a fixed factor. The comparisons among continuous parametric data were conducted by using t-test or Anova analysis, whereas the A Mann-Whitney U test or a Kruskal-Wallis test were used to analyze continuous nonparametric data. Comparisons between enumeration data ware conducted by Chi-Square or Fisher exact method. Survival comparison between groups was analyzed by using Log-rank analysis. *P* < 0.05 was considered to indicate statistical significance.

## Discussion

It has been confirmed that sepsis patients with severe thrombocytopenia have the higher mortality and the longer the duration of thrombocytopenia lasts, the worse the prognosis of these patients have. Platelets maybe a novel target to improve the prognosis of sepsis patients with severe thrombocytopenia. To date, there are three main platelet-elevating drugs in clinical practice, which are recombinant human interleukin-11(rhIL-11), thrombopoietin receptor agonist (TPO-RA) and rhTPO.

RhIL-11 is produced by recombinant DNA technology in *Escherichia coli*, which has shown effects on multiple hematopoietic cell types. And rhIL-11 could induce megakaryocyte maturation and stimulate megakaryocyte to increase PCs. In addition, rhIL-11 was involved in regulating the activity of non-hematopoietic cells, including regulating the growth of intestinal epithelial cells, osteoclast proliferation, host response and releasing inflammatory factors [33]. In hematological tumors patients with chemotherapy-induced thrombocytopenia, rhIL-11 could rapidly increase PCs, significantly reduce the incidence of bloodstream infection and improve the prognosis [34]. Recently, Wan B et al. reported that rhIL-11 could promote the recovery of PCs, reduce the expression of TNF-alpha and IL-6, alleviate inflammatory response, thus reducing the mortality of patients with thrombocytopenia [35]. However, rhIL-11 cannot increase the number of megakaryocytes and its clinical effect is relatively slow. Moreover, rhIL-11 itself is an inflammatory factor and has many adverse effects (including water and sodium retention, tissue edema, pleural effusion, acute pulmonary edema, arrhythmia), which limit the clinical application of rhIL-11 in sepsis. TPO-RAs (including eltrombopag or romiplostim) are another novel platelet-elevating drugs that bind to and activate the thrombopoietin (TPO) receptor, induce megakaryocyte progenitor proliferation by JAK2/STAT5 pathway, and finally increase PCs in chronic thrombocytopenia induced by immune, HCV infection or cirrhosis [36–38]. Until now, there is no report about TPO-RAs in sepsis treatment.

RhTPO specifically acts on the hematopoietic regulatory factors of megakaryocyte and promotes all stages of megakaryocyte formation. The drug is approved by the China State Food and Drug Administration for the treatment in patients with chronic immune or chemotherapy-related thrombocytopenia refractory to first-line therapy and no severe adverse effect has been reported. For sepsis, a retrospective study in elderly sepsis patients with thrombocytopenia reported that rhTPO increased PCs, reduced bleeding events, recovered organ function [39]. And rhTPO could rapidly restore PCs in 76.32% of patients with thrombocytopenia within 5 days, reduce the number of platelet transfusion and 28-day mortality in perioperative patients with abdominal infection [40]. However, there are the limitations in these two trials: 1) sepsis patients both were diagnosed by sepsis-1.0 standard; 2) PCs of whom was less than 100 × 10^9^/L; 3) the sample sizes were small and both of them were single-center non-randomized studies. The questions about the patients’ selecting, timing, effect and safety of rhTPO in sepsis are still unclear. Therefore, further studies in this field need to be explored, especially multi-center RCT studies.

In conclusion, RESCUE trial only focuses on sepsis patients with severe thrombocytopenia (PCs < 50 × 10^9^/L) and sepsis-3.0 standard will be used to diagnose which has the higher sensitivity than the other sepsis standards. To our knowledge, it is the first RCT study to explore the impact of rhTPO for severe thrombocytopenia in sepsis patients diagnosed by sepsis-3.0 standard. Furthermore, if the hypotheses are confirmed, RESCUE trial results will be of significant clinical value on the targeted therapy for sepsis patients with severe thrombocytopenia, and rhTPO probably becomes an effective rescue treatment for these sepsis patients except for platelet transfusion.

## Data Availability

The datasets used and/or analysed during the current study are available from the corresponding author on reasonable request.

## Abbreviations

APACHE II: Acute physiology and chronic health evaluation II
CRF: Case report form
CRP: C-reactive protein
DIC: Disseminated intravascular coagulation
PCs: Platelet counts
PI: Principle investigator
rhIL-11: Recombinant human interleukin-11
rhTPO: Recombinant human thrombopoietin
RRT: Renal replace therapy
SOFA: Sequential organ failure assessment
TNF-α: Tumor Necrosis Factor-alpha
TPO-RA: Thrombopoietin receptor agonist
